# Integrin Beta 1 Suppresses Multilayering of a Simple Epithelium

**DOI:** 10.1371/journal.pone.0052886

**Published:** 2012-12-21

**Authors:** Jichao Chen, Mark A. Krasnow

**Affiliations:** Department of Biochemistry and HHMI, Stanford University School of Medicine, Stanford, California, United States of America; Childrens Hospital Los Angeles, United States of America

## Abstract

Epithelia are classified as either simple, a single cell layer thick, or stratified (multilayered). Stratified epithelia arise from simple epithelia during development, and transcription factor *p63* functions as a key positive regulator of epidermal stratification. Here we show that deletion of integrin beta 1 (*Itgb1*) in the developing mouse airway epithelium abrogates airway branching and converts this monolayer epithelium into a multilayer epithelium with more than 10 extra layers. Mutant lung epithelial cells change mitotic spindle orientation to seed outer layers, and cells in different layers become molecularly and functionally distinct, hallmarks of normal stratification. However, mutant lung epithelial cells do not activate *p63* and do not switch to the stratified keratin profile of epidermal cells. These data, together with previous data implicating *Itgb1* in regulation of epidermal stratification, suggest that the simple-versus-stratified developmental decision may involve not only stratification inducers like *p63* but suppressors like *Itgb1* that prevent simple epithelia from inappropriately activating key steps in the stratification program.

## Introduction

Epithelia cover the external and internal surfaces of all multi-cellular organisms, and they separate compartments by forming physical and physiological barriers. One of the most fundamental distinctions among epithelia is whether they are simple, a cell monolayer with each cell attached to the underlying basement membrane, or stratified, in which there are two or more layers with just the basally located cells attached to the basement membrane [1]. The non-basal cell layers in a stratified epithelium provide additional mechanical support to the tissue and may undergo further molecular and functional specialization, such as cornification [1].

Stratified epithelia appear to arise from simple epithelia during development, and this stratification process has been best studied in the epidermis. During stratification, epidermal progenitor cells switch from symmetric cell division, where the mitotic spindle orients parallel to the basement membrane and gives rise to functionally equivalent daughter cells, to predominantly asymmetric cell division, where the mitotic spindle orients perpendicular to the basement membrane and generates a self-renewing basal cell and a differentiating supra-basal cell; this transition depends on protein complexes controlling cell polarity and spindle positioning [2], [3], [4]. In addition to these cellular changes, epidermal progenitor cells switch their keratin profile by down-regulating keratin genes specific for simple epithelia (*Krt8* and *Krt18*), and activating ones specific for stratified epithelia (*Krt5* and *Krt14*) [5]. Loss and gain-of-function studies have shown that transcription factor *p63* is a key positive regulator of the cellular and molecular changes during stratification [6], [7], [8]. Little is known about the simple-versus-stratified decision in other developmental contexts.

The bronchial tree of the mouse lung is a hierarchical tubular network, which initiates as simple protrusions from the embryonic foregut and undergoes an extensive, yet highly organized branching program [9], [10]. Each branch consists of a simple monolayer epithelium that buds from a parental branch and remains a monolayer as it undergoes one of three branching subroutines to generate the thousands of branches of the bronchial tree [10]. Gene expression and mutant analyses have identified a number of important signaling pathways, including fibroblast growth factor (FGF), Hedgehog and WNT pathways, involved in reciprocal interactions between the airway epithelium and the surrounding mesenchyme during airway branching [9]. Much less is known about the cellular events, such as cell division, movement, polarization and monolayer organization and maintenance, and the cellular effectors that these signaling pathways control to generate new branches.

Integrins are α/β heterodimeric transmembrane receptors for ligands in the extracellular matrix, and they are essential for both cell adhesion and activation of intracellular signaling pathways [11]. Integrins have long been implicated in lung branching morphogenesis because multiple integrins are expressed in the developing airway epithelium and surrounding mesenchyme [12], [13], [14], and blocking peptides reduce airway branching in culture [15]. Furthermore, double mutants in integrin alpha 3 (*Itga3*) and alpha 6 (*Itga6*) have severely hypoplastic lungs, although branching still proceeds [16]. However, it is not clear from these studies to what extent, in which cell type(s), and by what cellular mechanisms airway development is dependent on integrin signaling.

Here we show that conditional deletion throughout the developing mouse lung epithelium of *Itgb1*, which is the major isoform of the eight beta integrin subunits and forms 12 of the 24 known integrin α/β heterodimers [11], abrogates airway branching and converts the affected region into a multilayer epithelium with more than 10 extra layers. This ectopic multilayering is accompanied by some of the cellular events of epidermal stratification, and generates molecularly distinct layers with different fates, hallmarks of epithelial stratification. However, *p63* and the full epidermal stratification program are not activated. These results, together with previous data implicating *Itgb1* in the regulation of epidermal stratification, suggest that *Itgb1* may serve to suppress key steps in stratification.

## Results

### Formation of a multilayer lung epithelium following deletion of *Itgb1*

**Figure 1:**
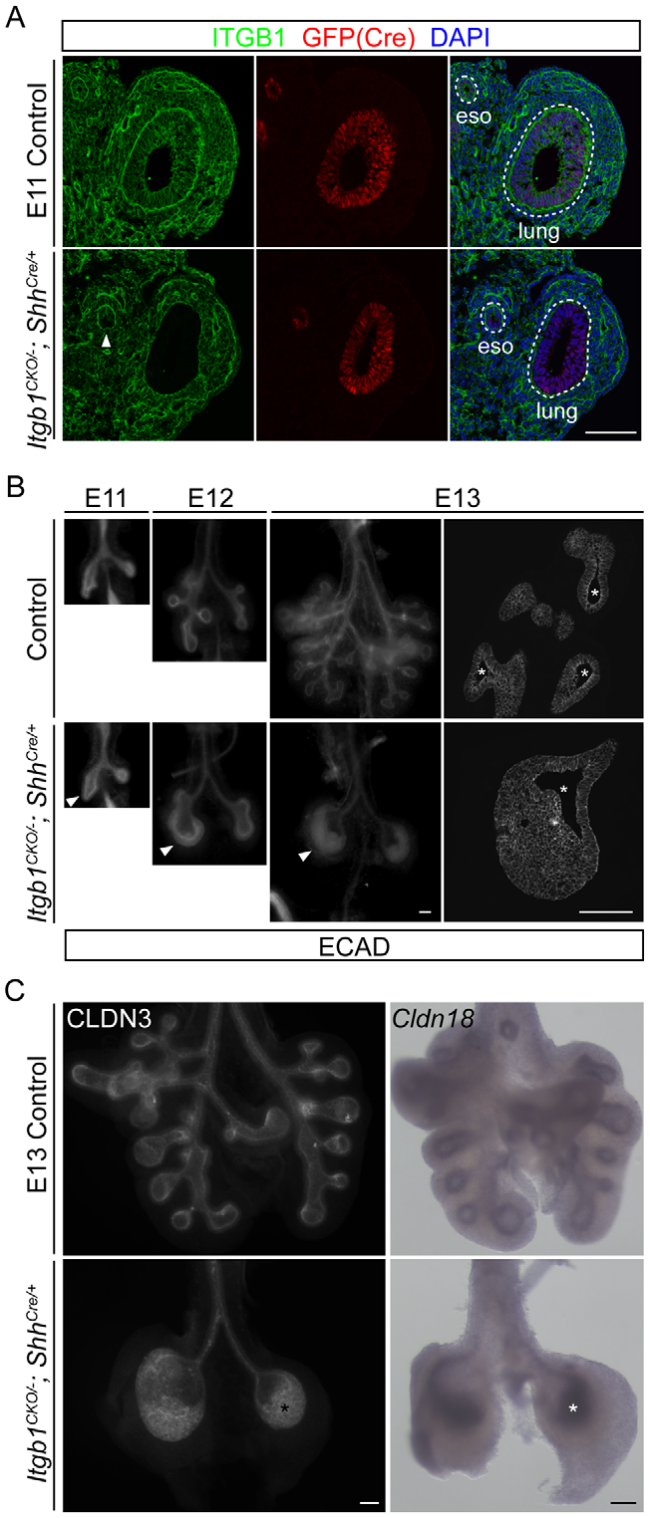
Epithelial inactivation of *Itgb1* inhibits branching and leads to a multilayer epithelium during lung development. (A) Section immunostaining showing complete loss of ITGB1 specifically in the lung epithelial cells, but not the surrounding mesenchymal cells as early as embryonic day 11 (E11) in the *Itgb1^CKO/−^; Shh^Cre/+^* mutant. Sections were co-stained for GFP to visualize cells expressing the GFP-CRE fusion protein under the control of the *Shh* promoter, and nuclei were counter-stained with 4′,6-diamidino-2-phenylindole (DAPI). The arrowhead indicates partial loss of ITGB1 in the ventral half of the esophagus epithelium in the *Itgb1^CKO/−^; Shh^Cre/+^* mutant. The dashed lines demarcate the basal side of the lung and esophagus (eso) epithelia. Scale bar, 100 um. (B) Whole-mount (left three columns) and section (right column) immunolocalization of E-Cadherin (ECAD) showing the inhibition of branching and progressive formation of a multilayer lung epithelium (arrowheads) at sequential embryonic days (E11, E12, E13) in the *Itgb1^CKO/−^; Shh^Cre/+^* mutant. Because of the three-dimensional structure of the lung, the epithelium can artificially appear multilayered in tangential sections through the epithelium. The number of epithelial layers can only be accurately assessed in regions of sections where the lumenal space (asterisks) is visible; note that there are more than 10 cell layers in the E13 mutant lung. Scale bar, 100 um. (C) Whole-mount immunostaining (left panels) and *in situ* hybridization (right panels) of E13 lungs showing that cells (asterisks) of the multilayer epithelium in the *Itgb1^CKO/−^; Shh^Cre/+^* mutant lung maintain expression of epithelial markers CLDN3 (left panels) and *Cldn18* (right panels). Scale bar, 100 um.

*Itgb1* is uniformly expressed throughout the developing lung epithelium, as well as the mesenchyme, the methothelium and the vasculature (Figure 1A and S1A). Homozygous *Itgb1* null mutants die shortly after implantation [17], [18], so to assess *Itgb1* function in the developing lung epithelium we used a conditional *Itgb1* allele with the third exon flanked by LoxP sites [19], and inactivated it using a *Shh^Cre^* knock-in allele that has been shown to induce specific recombination of reporter genes throughout the lung epithelium [20]. The CRE recombinase is expressed as a GFP-CRE fusion protein under the control of the *Shh* promoter [21]. We confirmed the epithelium-specific expression of the GFP-CRE fusion protein in E11 lungs by immunostaining for GFP (Figure 1A). Immunostaining for ITGB1 showed that the protein was specifically lost throughout the lung epithelium, and the loss was complete as early as E11 (Figure 1A and S1A).

*Itgb1^CKO/−^; Shh^Cre/+^* mutants were viable and were obtained at Mendelian ratios up to embryonic day 19 (E19), but died shortly after birth in respiratory distress. Anatomical examination of newborn *Itgb1^CKO/−^; Shh^Cre/+^* mutants revealed a small and severely malformed lung with just left and right primary bronchi ( Figure S1B). To pinpoint the earliest lung defects, we analyzed E11 to E19 lungs by whole-mount immunostaining for E-Cadherin, an epithelial marker. The left and right primary bronchi initiated normally but subsequent branches failed to form (Figure 1B), and no expression was detected of surfactant gene *Sftpc*, a gene normally restricted to the branching regions of the lung (Figure S2A). Despite the complete abrogation of branching, the *Itgb1* mutant epithelial cells expressed the lung lineage marker NKX2.1 (data not shown) and showed the normal pattern of proximal (SOX2) and distal (SOX9) epithelial markers (Figure S2B).

Unlike most other mutants with branching defects, where the lung epithelium remains a monolayer [9], the epithelium in *Itgb1^CKO/−^; Shh^Cre/+^* mutants formed a striking multilayer structure, 10 or more cell layers thick (Figure 1). The *Itgb1* mutant epithelium started to thicken at E12 and peaked between E13 and E14. After E14, the thickness began to decrease (Figure S1B), at least in part due to apoptosis (see below). Interestingly, despite the uniform expression of *Itgb1* throughout the lung epithelium (Figure 1A and S1A), this multilayering was restricted to the regions of the lung that normally undergo branching, and was not observed in the extra-pulmonary, non-branching regions including the trachea.

### The mutant epithelium maintains expression of epithelial markers

One way epithelia form multilayered structures is through an epithelial-to-mesenchymal transition (EMT), a process in which epithelial character and marker expression are lost, as has been observed in both normal development and epithelial cancers [22], [23], [24]. Such epithelial cells down-regulate expression of E-Cadherin, lose contact with neighboring epithelial cells and eventually detach from the original epithelial sheet. However, all of the *Itgb1* mutant lung epithelial cells still express normal levels of E-Cadherin (Figure 1B) and other genes encoding epithelial junction proteins including Claudin 3 and Claudin 18 (Figure 1C), implying that EMT is not the mechanism of multilayering. Instead, we show below that the multilayering process in the *Itgb1* mutant lung shares some features of normal epidermal stratification.

### Change in mitotic spindle orientation and partial loss of apical-basal polarity in the mutant epithelium

**Figure 2:**
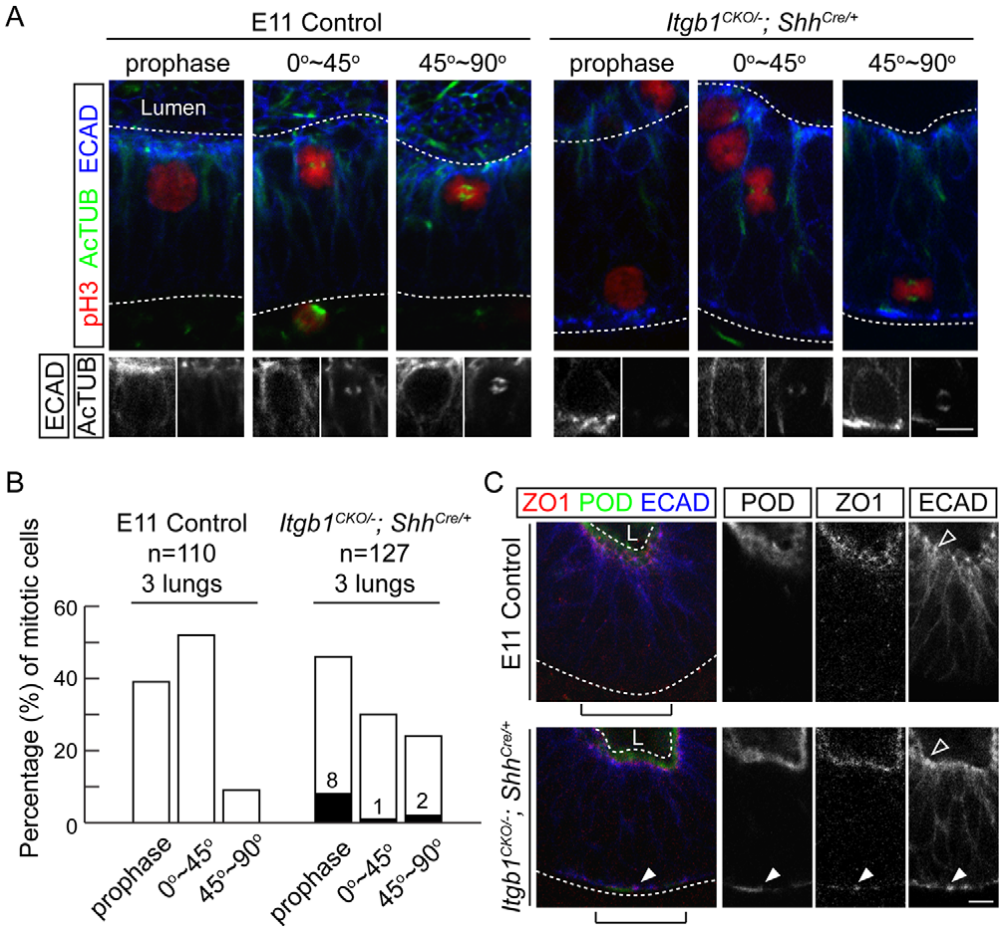
*Itgb1^CKO/−^; Shh^Cre/+^* mutant lung epithelial cells reorient mitotic spindles and show partial loss of apical-basal polarity. (A) Confocal sections of whole-mount E11 lungs immunostained for markers of mitosis (phospho-Histone 3, pH3), mitotic spindles (acetylated Tubulin, AcTUB) and the epithelium (E-Cadherin, ECAD). Representative images are shown for mitotic cells with no obvious mitotic spindle (prophase) and with the mitotic spindle orientated between 0 to 45 degrees (0°∼45°) or 45 to 90 degrees (45°∼90°) relative to the lumenal surface of the epithelium. Mitotic cells in the control lung are exclusively localized within one nuclear diameter of the lumenal surface of the epithelium. The *Itgb1^CKO/−^; Shh^Cre/+^* mutant lung contains mitotic cells localized away from the lumenal surface and sometimes on the basal surface of the epithelium. Grey scale images of the ECAD and AcTUB staining are shown below for the mitotic cells of interest. Note the intense ECAD puncta associated with the basally located *Itgb1* mutant nuclei. The upper and lower dashed lines demarcate the lumenal (Lumen) and basal side of the epithelium, respectively. Scale bar, 10 um. (B) Quantification of the percentage of cells in each category in (*A*) out of 110 (control) and 127 (mutant) mitotic cells from 3 E11 lungs. The filled columns and the associated numbers indicate the percentage of mitotic cells that are located more than one nuclear diameter from the lumenal surface of the epithelium in the mutant. The control and mutant epithelia are significantly different in the distributions of mitotic cells between the 0°∼45°and 45°∼90° categories (52% and 9% in the control versus 30% and 24% in the mutant, p = 0.0004, Chi-square test), and in the number of mitotic nuclei located more than one nuclear diameter away from the lumenal surface of the epithelium (0 out of 110 in the control versus 14 out of 127 in the mutant, p = 0.0001, Fisher's exact test). (C) Confocal sections of whole-mount immunostained E11 lungs reveal basally located (arrowheads) markers of apical membrane (Podocalyxin-like, POD) and tight junction (ZO1) in the *Itgb1^CKO/−^; Shh^Cre/+^* mutant. In the control lung, cell junction protein E-Cadherin (ECAD) is localized between cells on the basal-lateral side of the epithelium and most concentrated as puncta (hollow arrowhead) close to the lumenal side. In the *Itgb1^CKO/−^; Shh^Cre/+^* mutant, additional ECAD puncta are localized to the basal side of the epithelium (arrowhead). These ectopic ECAD puncta are not obvious at low magnification or on sections (e.g., Figure 1B). The upper and lower dashed lines demarcate the lumenal (L) and basal side of the epithelium, respectively. Grey scale images are shown of the bracketed regions. Scale bar, 10 um.

One critical step in stratification of the epidermis is a switch in the orientation of cell division from primarily (∼90%) parallel to the basement membrane, which are functionally symmetrical divisions that promote self-renewal of basal cells, to predominantly (∼75%) perpendicular cell divisions [2]. These perpendicular divisions are asymmetric and generate supra-basal daughter cells that promote stratification [3]. To test if asymmetric cell divisions occur during formation of the multilayered *Itgb1* mutant epithelium, we examined mitotic spindle orientation at E11, when the primary bronchi have just formed and before there are any obvious morphological abnormalities. We imaged mitotic spindles by confocal microscopy of whole-mount E11 lungs immunostained for phospho-Histone 3 and acetylated Tubulin (Figure 2A), which allowed visualization of spindles in three dimensions and avoided biases due to physical sectioning. In control lungs, the spindles of most (>85%) mitotic epithelial cells were oriented parallel to the lumenal surface, whereas in the *Itgb1* mutant lung epithelium there was a nearly equal mix of mitotic cells with spindles oriented parallel (55%) versus perpendicular (45%) to the lumenal surface (p = 0.0004) (Figure 2B). There was no significant difference in either the number of mitotic cells or the percentage of mitotic cells in prophase between the control and *Itgb1* mutant lung epithelia (Figure 2B). We conclude that mitotic spindle orientation is altered in the *Itgb1* mutant lung, such that perpendicular (asymmetric) cell divisions, like those that seed outer layers during epidermal stratification, become prominent.

The observed changes in mitotic spindle orientation in the *Itgb1* mutant epithelium were associated with alterations in apical-basal polarity. Apical membrane protein Podocalyxin-like (PODXL), tight junction protein 1 (ZO1) and adherent junction protein ECAD (Figure 2C), which are normally either exclusively localized (PODXL and ZO1) or highly enriched as puncta (ECAD) on the lumenal (apical) side of the monolayer epithelium, all ectopically localized to the basal surface of the *Itgb1* mutant lung epithelium, in addition to their usual apical localization (Figure 2C). We also found that the nuclei of some (∼10%) mitotic cells in the *Itgb1* mutant lung epithelium were positioned more than one nuclear diameter away from the lumenal (apical) surface of the epithelium, whereas mitotic cell nuclei in the control epithelium were always located within one nuclear diameter, implying a defect in interkinetic nuclear migration [25] (Figure 2A and B; p = 0.0001). In extreme cases, the nuclei of *Itgb1* mutant mitotic cells were located at the basal surface of the epithelium, and these basal nuclei were always associated with intense ectopic accumulation of E-Cadherin puncta (Figure 2A). Such distribution of apical proteins is reminiscent of that of E-Cadherin in the suprabasal cells during epidermal stratification [26]. Thus, loss of *Itgb1* leads to reorientation of mitotic spindles and partial loss of apical-basal polarity in a normally simple epithelium.

### Differences among layers in the mutant epithelium

**Figure 3:**
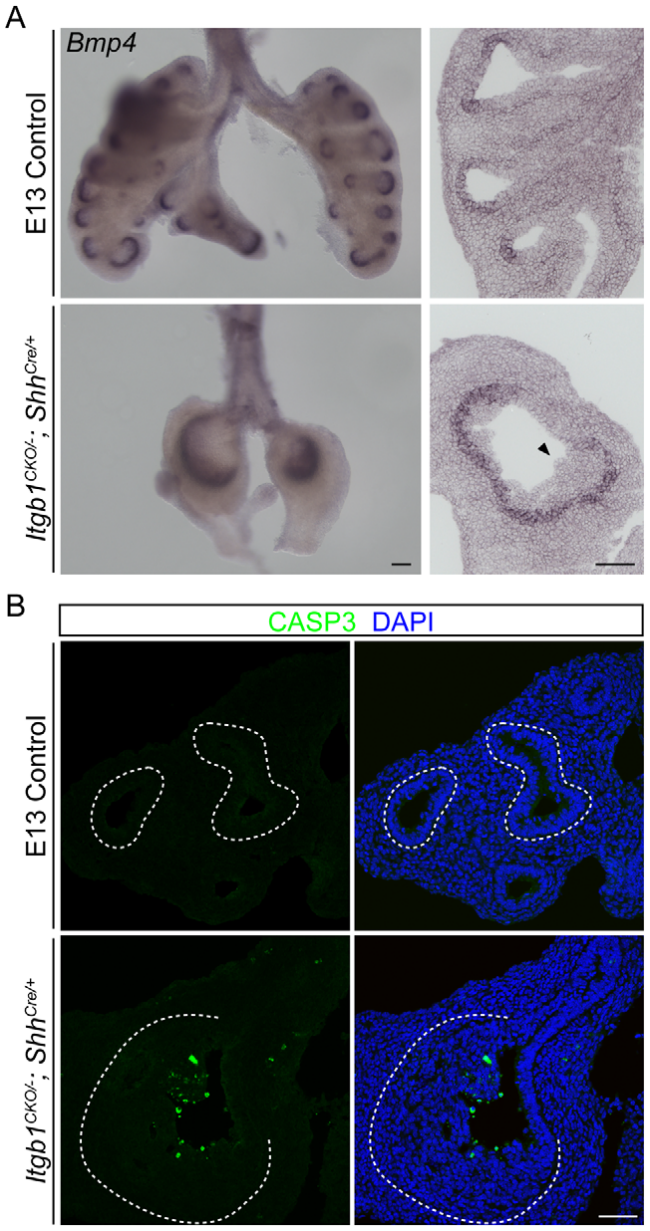
Differences among cells in different layers of the multilayer *Itgb1* mutant lung epithelium. (A) Whole-mount (left column) and section (right column) *in situ* hybridization of E13 lungs showing that the expression of *Bmp4* remains restricted to the distal branching epithelium in the *Itgb1^CKO/−^; Shh^Cre/+^* mutant, and that only cells on the basal side, but not on the lumenal side (arrowhead), of the multilayer epithelium in the *Itgb1^CKO/−^; Shh^Cre/+^* mutant lung express *Bmp4*. Scale bar, 100 um. (B) Section immunostaining of E13 lungs showing that cleaved Caspase-3 (CASP3), a marker of apoptosis, is preferentially expressed by cells located on the lumenal side of the multilayer epithelium in the *Itgb1^CKO/−^; Shh^Cre/+^* mutant lung. Nuclei were counter-stained with DAPI. The dashed lines demarcate the basal side of the lung epithelium. Scale bar, 50 um.

Another prominent feature of stratified epithelia, including the epidermis, is the diversification of layers, with cells in the basal layer renewing the epithelium and cells in the suprabasal layers undergoing special keratinization and sometimes becoming cornified [1]. Molecular and cellular differences were also found among layers of the *Itgb1* mutant lung epithelium. Although most genes examined, including E-Cadherin (Figure 1B), Claudin 3 and Claudin 18 (Figure 1C), were expressed uniformly across the multilayer epithelium, expression of *Bmp4* and *Spry2* was restricted to cells in just the basal layer (Figure 3A and S3). Because expression of these genes is induced by mesenchyme-derived signals, those signals must not be able to reach or activate cells in the suprabasal layers. Cells in the layers farthest from the mesenchyme also appear to be functionally distinct because they selectively undergo apoptosis, as shown by staining for cleaved Caspase-3 (Figure 3B). In contrast, only rare cells in layers closer to the mesenchyme, or in the control epithelium, expressed the apoptotic fate. This may reflect the diffusion limit of a trophic signal, or activation of a senescence program as cells move away from the basal layer and are ultimately shed. We conclude that there are molecular and functional differences among layers of the *Itgb1* mutant lung epithelium.

### The mutant epithelium does not activate the epidermal stratification expression program

**Figure 4:**
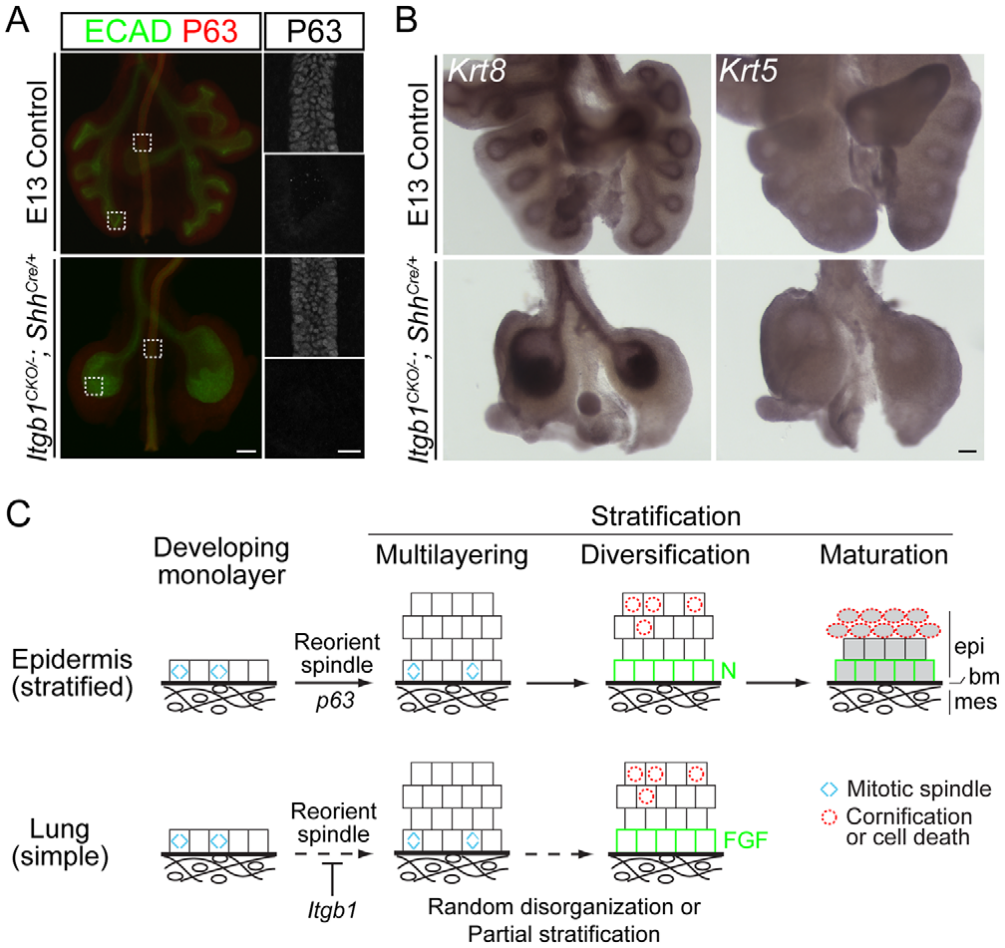
The multilayer epithelium in the *Itgb1^CKO/−^; Shh^Cre/+^* mutant lung expresses markers of simple epithelium. (A) Stereoscope (left; scale bar, 100 um) and confocal (right; scale bar, 20 um) images of E13 whole-mount lungs immunostained for E-Cadherin (ECAD) and P63. The boxed regions in the left panels are imaged by confocal microscopy and shown in the right panels. P63 is expressed in the esophagus (top), but not in the branching regions of the lungs (bottom), of both control and *Itgb1^CKO/−^; Shh^Cre/+^* mutant embryos. (B) Whole-mount *in situ* hybridization shows that the epithelia of both control and *Itgb1^CKO/−^; Shh^Cre/+^* mutant lungs express a marker of simple epithelium (*Krt8*), but not a marker of stratified epithelium (*Krt5*) at E13. Scale bar, 100um. (C) A three-part model of epithelial stratification comparing epidermis and lung. *Layer formation*: regulation of mitotic spindle orientation (blue carets) drives formation of a multilayer epithelium; *layer diversification*: cell autonomous and/or non-autonomous signals lead to molecular and functional specialization of cells of different layers (green squares, cells with low Notch (N) pathway activity (epidermis) or high FGF pathway activity (lung); red dashed circles, cells undergoing cell death and cornification (epidermis) or cell death alone (lung)); *layer maturation*: activation of genes such as keratin genes specific for stratified epithelia and suppression of ones for simple epithelia leading to differentiation of the layers (shaded cells). Although the maturation step is shown last, the keratin profile switch in epidermis initiates in the monolayer and continues throughout stratification. In the absence of *Itgb1*, the normally simple lung epithelium may be in a random, disorganized state. However, the *Itgb1* mutant epithelium shares two of the three features of epidermal stratification, suggesting that it undergoes partial stratification. epi, epithelium; bm, basement membrane; mes, mesenchyme.

We tested whether the *Itgb1* mutant lung epithelium activated the epidermal stratification gene expression program. We were particularly interested in *p63*, a transcription factor that controls epidermal stratification, because it can initiate stratification when ectopically expressed in late gestational and adult lungs [8]. However, no P63 was detected in the branching regions of *Itgb1^CKO/−^; Shh^Cre/+^* mutant or control lungs either by whole-mount or section immunostaining (Figure 4A and data not shown). Furthermore, the *Itgb1* mutant epithelium maintained expression of the simple epithelium markers, keratin genes *Krt8* and *Krt18*, and did not turn on the corresponding stratified epithelium markers *Krt5* and *Krt14* (Figure 4B and S4). Thus, although the multilayer *Itgb1* mutant lung epithelium shows some of the cellular and molecular features of a stratified epithelium, it does not turn on *p63* and execute the full epidermal stratification program.

## Discussion

We have shown that conditional deletion of integrin *Itgb1* throughout the developing airway epithelium abolishes airway branching and converts this monolayer epithelium into a multilayer structure with more than 10 extra layers. Although this extensive multilayering might simply be a disordered structure resulting from loss of ITGB1-mediated cell adhesion or polarity, the mutant epithelium exhibited three other features of stratified epithelia beyond multilayering. First, mutant cells maintained expression of epithelial markers and the epithelium remained associated with the underlying basement membrane. Second, the multilayered structure displayed a higher level organization with molecular and functional distinctions among the layers: basal layer cells expressed *Bmp4* and *Spry2*, whereas cells in layers farthest from the mesenchyme selectively activated the apoptotic program. Third, mitotic spindles reoriented from almost exclusively parallel to the basement membrane in wild type to a mixture of parallel and perpendicular divisions in the mutant lung epithelium, similar to the spindle reorientation previously observed during epidermal stratification where perpendicular divisions that send daughter cells away from the basement membrane become prominent [2]. Importantly, downregulation of *Itgb1* expression appears to be part of the normal stratification program: *Itgb1* is expressed in basal cells of the epidermis but absent from their daughter cells that populate the suprabasal layers [26], and complete loss of *Itgb1* in basal cells leads to hyperthickened skin [27], reminiscent of the *Itgb1* mutant lung phenotype.

These results lead us to propose that *Itgb1* suppresses multilayering of epithelia, and it does so by promoting symmetric cell divisions (parallel to the basement membrane) such that both daughters from a division remain in the epithelial plane and attached to the basement membrane. Deletion or downregulation of *Itgb1* alters apical-basal polarity and the cell division plane, so perpendicular cell divisions becomes prominent, each leaving one daughter in contact with the basement membrane while the other is sent outward to seed suprabasal layers. According to this model, the monolayer structure of the airway epithelium is not simply a default state, but rather is actively maintained by *Itgb1*. The model further implies that downregulation of *Itgb1* expression and function is critical for normal stratification, a proposition that can be tested by maintaining expression during epidermal stratification with a constitutive *Itgb1* transgene.

If this view of *Itgb1* is correct, then the simple-versus-stratified epithelial decision is controlled not just by expression of stratification activators like *p63*, but by the balance between activators and stratification suppressors like *Itgb1*. Indeed, neither loss of *Itgb1* or expression of *p63* activates a full stratification program, at least in the lung. Loss of *Itgb1* in the developing bronchial epithelium led to multilayer formation and some layer diversification but not induction of *p63* and stratified keratin genes or suppression of monolayer keratins. Ectopic overexpression of *p63* in the lung epithelium induces a stratified keratin profile and formation of extra layers, but the number of extra layers is limited and there is no obvious specialization among layers [8].

We suggest the following three-part model for stratification (Figure 4C). First, downregulation of *Itgb1* expression or function alters cell polarity and mitotic spindle orientation to generate daughter cells that are not attached to the basement membrane, promoting formation of additional cell layers (“layer formation”). Second, cells in different layers undergo distinct molecular and functional specializations (“layer diversification”), for example FGF signaling in the basal cell layer and apoptosis in the non-basal cell layer in the developing lung epithelium, and low Notch activity and self-renewal in the basal cell layer and cornification in the non-basal cell layer in the epidermis. These molecular and functional specializations may be a result of intrinsic cell differences, such as Notch signaling [3], and/or differential extrinsic signals from the basement membrane or underlying mesenchyme. Third, *p63* drives “layer maturation”, for example by suppressing keratin genes specific for simple epithelium and activating keratin and other genes specific for stratified epithelium.

If the monolayer structure of the airway epithelium is not simply a default state as widely presumed, perhaps stratification – with the additional mechanical support and a greater barrier function it provides – was the ancestral condition of epithelia. Negative regulators like *Itgb1* could then have evolved to suppress stratification and generate a monolayer that facilitates physiological movement of chemicals, such as gas transport across the lung alveolar epithelium or nutrient absorption across the intestine, and developmental cell movements and morphogenesis. Indeed, loss of *Itgb1* and another beta-integrin have been reported to cause at least a limited degree of multilayering in several other animal tissues [27], [28], [29]. It will be important to elucidate the full complement of negative and positive regulators of simple and stratified epithelia and how they interact during development, homeostasis, and diseases including cancer where normal control of the simple-versus-stratified developmental decision is lost.

## Materials and Methods

### Mice

Mice carrying the *Itgb1* conditional allele (*Itgb1^CKO^*) carrying Cre recombination sites flanking exon 3 of *Itgb1* [19] were mated to female *HPRT^Cre/+^* mice [30] to generate mice carrying a germ line *Itgb1* null allele (*Itgb1^−^*), which were subsequently crossed to *Shh^Cre/+^* mice [21]. *Itgb1* lung epithelium mutants (*Itgb1^CKO/−^; Shh^Cre/+^* ) and phenotypically wild type littermates (control), including *Itgb1^CKO/+^; Shh^Cre/+^* or *Itgb1^CKO/−^* or *Itgb1^CKO/+^*, were generated by crossing *Itgb1^+/−^; Shh^Cre/+^* males with *Itgb1^CKO/CKO^* females. Mice carrying *Itgb1^CKO^, HPRT^Cre^* and *Shh^Cre^* alleles were genotyped according to protocols from the Jackson Laboratory (Bar Harbor, ME). The *Itgb1^−^* allele was genotyped using primers 5′-CGCAGAACAATAGGTGCTGAAATTAC-3′ and 5′-CCACAACTTTCCCAGTTAGCTCTC-3′.

### Tissue preparation and immunostaining

Embryos were isolated from timed pregnant mice with the day the vaginal plug was observed designated as E1. Lungs were dissected and fixed with 4% paraformaldehyde in phosphate-buffered saline (PBS) for 1 hr at 4°C. For section immunostaining, the fixed lungs were cryoprotected in PBS with 20% sucrose at 4°C overnight and then embedded in Optimal Cutting Temperature Compound (OCT; Tissue-Tek, Tokyo, Japan). Immunostaining of frozen sections was carried out essentially as described [31]. For whole-mount immunostaining, the lungs were incubated with blocking buffer (PBS with 5% normal goat or donkey serum and 0.3% Triton X-100), and then incubated with primary antibodies in blocking buffer at 4°C overnight. The following day, the lungs were washed in wash buffer (PBS with 1% Triton X-100 and 1% Tween-20) for 6 hr at room temperature and then incubated with secondary antibodies diluted in blocking buffer at 4°C overnight. The lungs were then washed as described above and mounted with the ventral side facing up. Images were captured on a fluorescence stereoscope (MZ16FA, Leica) or a confocal microscope (SP2, Leica). The following antibodies were used: rat anti-ITGB1 (1∶250, MAB1997, Millipore), chicken anti-GFP (1∶500, AB13970, Abcam), rat anti-ECAD (1∶500, 131900, Invitrogen), rabbit anti-cleaved Caspase-3 (1∶250, #9661, Cell Signaling Technology), rabbit anti-ZO1 (1∶50, 187430, Invitrogen), goat anti-PODXL (1∶250, AF1556, R&D Systems), mouse anti-phosphoHistone 3 IgG1 (1∶1000, 05–806, Millipore), mouse anti-acetylated Tubulin IgG2b (1∶1000, T6793, Sigma), rabbit anti-P63 (1∶100, sc-8343, Santa Cruz Biotechnology) and rabbit anti-CLDN3 (1∶250, RB-9251, LabVision).

### *In situ* hybridization

*In situ* hybridization was performed essentially as described [32]. Digoxigenin-labeled riboprobes were transcribed with T7 RNA polymerase from the indicated cDNAs, which were isolated as cloned PCR products derived from mouse embryonic lung RNA. To minimize experimental variation, control and *Itgb1* mutant lungs were processed in the same tube for each riboprobe throughout the *in situ* experiment. Images were captured on a Leica MZ12 dissecting scope or a Zeiss Axiophot microscope.

### Quantification of mitotic spindle orientation

Whole-mount immunostained E11 lungs were mounted with ventral side up, and Z stack images of the entire lung epithelium were collected at 2 um intervals with a 40× oil objective (N.A. = 1.25) on a Leica TCS SP2 confocal microscope. Due to the three-dimensional structure of the lung and the limited resolution of confocal images in the Z dimension, only images showing longitudinal cross-sections of epithelial tubes with visible lumenal space were used for the quantification of mitotic spindle orientation. Only the branching regions of the lung epithelium were included in the quantification. Mitotic cells within the lung epithelium were identified based on co-expression of phospho-Histone 3 and E-Cadherin. The orientations of mitotic spindles were determined by the bi-polar distribution of acetylated Tubulin, and assigned to one of two categories, 0 to 45 degrees (0°∼45°) or 45 to 90 degrees (45°∼90°), by comparing the orientation of the mitotic spindle with its nearest lumenal surface of the lung epithelium. Accurate angular measurement of mitotic spindle orientation was not possible because of the three-dimensional curvature of the lumenal surface of the lung epithelium. Mitotic cells without clear bi-polar distribution of acetylated-Tubulin were considered to be in prophase.

## Supporting Information

Figure S1 (A) Section immunostaining showing complete loss of ITGB1 specifically in the lung epithelial cells, but not the surrounding mesenchymal cells at E13 in the *Itgb1^CKO/−^; Shh^Cre/+^* mutant. Nuclei were counter-stained with 4′,6-diamidino-2-phenylindole (DAPI). Asterisks indicate lumenal space. Scale bar, 100 um. (B) Whole-mount ECAD immunostaining of E16 control (left panel), E16 (middle panel) and postnatal day (P) 0 (right panel) *Itgb1^CKO/−^; Shh^Cre/+^* mutant lungs. Very few epithelial cells accumulate in the lumen of the *Itgb1* mutant lung at E16 (asterisk), compared to the multilayer mutant epithelium at E13 (Figure 1B). At P0, the *Itgb1* mutant lung consists of dilated left and right main bronchi with small alveolus-like structures attached (arrowhead, inset, scale bar, 40 um). Scale bar, 400 um.
(TIF)

Figure S2 (A) Whole-mount *in situ* hybridization of E13 lungs shows that the *Itgb1* mutant lung does not express *Sftpc*, a gene restricted to the branching regions of the control lung. Scale bar, 100 um. (B) Whole-mount immunostaining of E-Cadherin (ECAD), SOX9 and SOX2 of E13 lungs showing normal distribution of distal (SOX9) and proximal (SOX2) epithelial markers in the *Itgb1* mutant lung. The mesenchymal SOX9 staining surrounding the extra-pulmonary airways (arrowhead) is from cartilage precursor cells. Scale bar, 200 um.
(TIF)

Figure S3 **Whole-mount** ***in situ*** **hybridization of** ***Spry2*** **in E13 lungs.** Expression of *Spry2* is restricted to the distal branching epithelium in the *Itgb1^CKO/−^; Shh^Cre/+^* mutant, like that of *Bmp4* (Figure 4A), Cells on the lumenal side (arrowhead) of the multilayer mutant epithelium do not express *Spry2*. Scale bar, 100 um.
(TIF)

Figure S4 **Whole-mount** ***in situ*** **hybridization shows that the epithelia of both control and** ***Itgb1^CKO/−^; Shh^Cre/+^*** **mutant lungs express a marker of simple epithelium (** ***Krt18*** **), but not a marker of stratified epithelium (** ***Krt14*** **) at E12.** Scale bar, 100 um.
(TIF)
