# Supplementary figures and images for: Integrin Beta 1 Suppresses Multilayering of a Simple Epithelium
Source: PLoS One. 2012 Dec 21;7(12):e52886. doi: 10.1371/journal.pone.0052886 (PMC3528644; doi:10.1371/journal.pone.0052886)

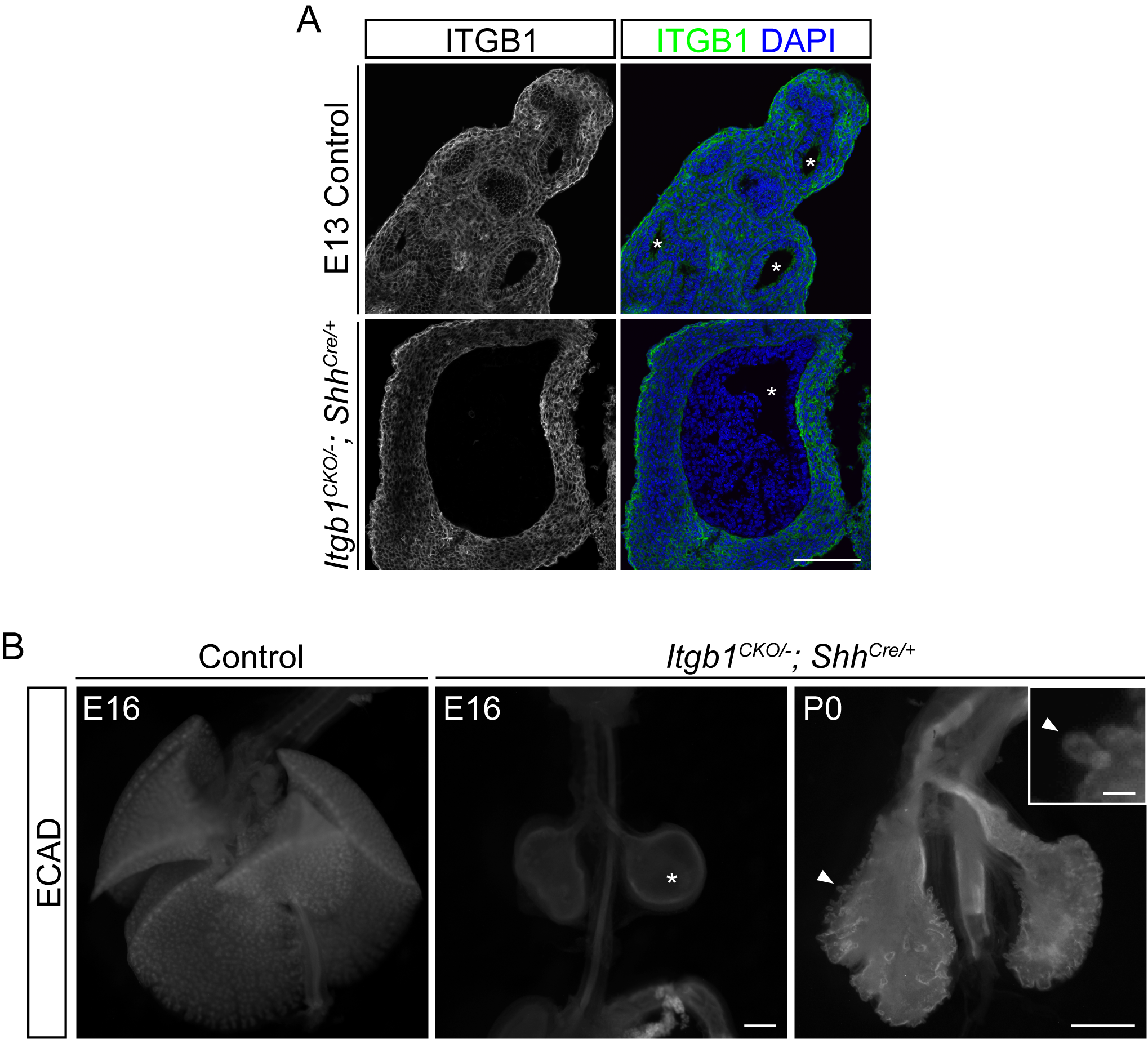

Supplement: Figure S1 — (A) Section immunostaining showing complete loss of ITGB1 specifically in the lung epithelial cells, but not the surrounding mesenchymal cells at E13 in the Itgb1CKO/−; ShhCre/+ mutant. Nuclei were counter-stained with 4′,6-diamidino-2-phenylindole (DAPI). Asterisks indicate lumenal space. Scale bar, 100 um. (B) Whole-mount ECAD immunostaining of E16 control (left panel), E16 (middle panel) and postnatal day (P) 0 (right panel) Itgb1CKO/−; ShhCre/+ mutant lungs. Very few epithelial cells accumulate in the lumen of the Itgb1 mutant lung at E16 (asterisk), compared to the multilayer mutant epithelium at E13 (Figure 1B). At P0, the Itgb1 mutant lung consists of dilated left and right main bronchi with small alveolus-like structures attached (arrowhead, inset, scale bar, 40 um). Scale bar, 400 um. (TIF) [file pone.0052886.s001.tif]

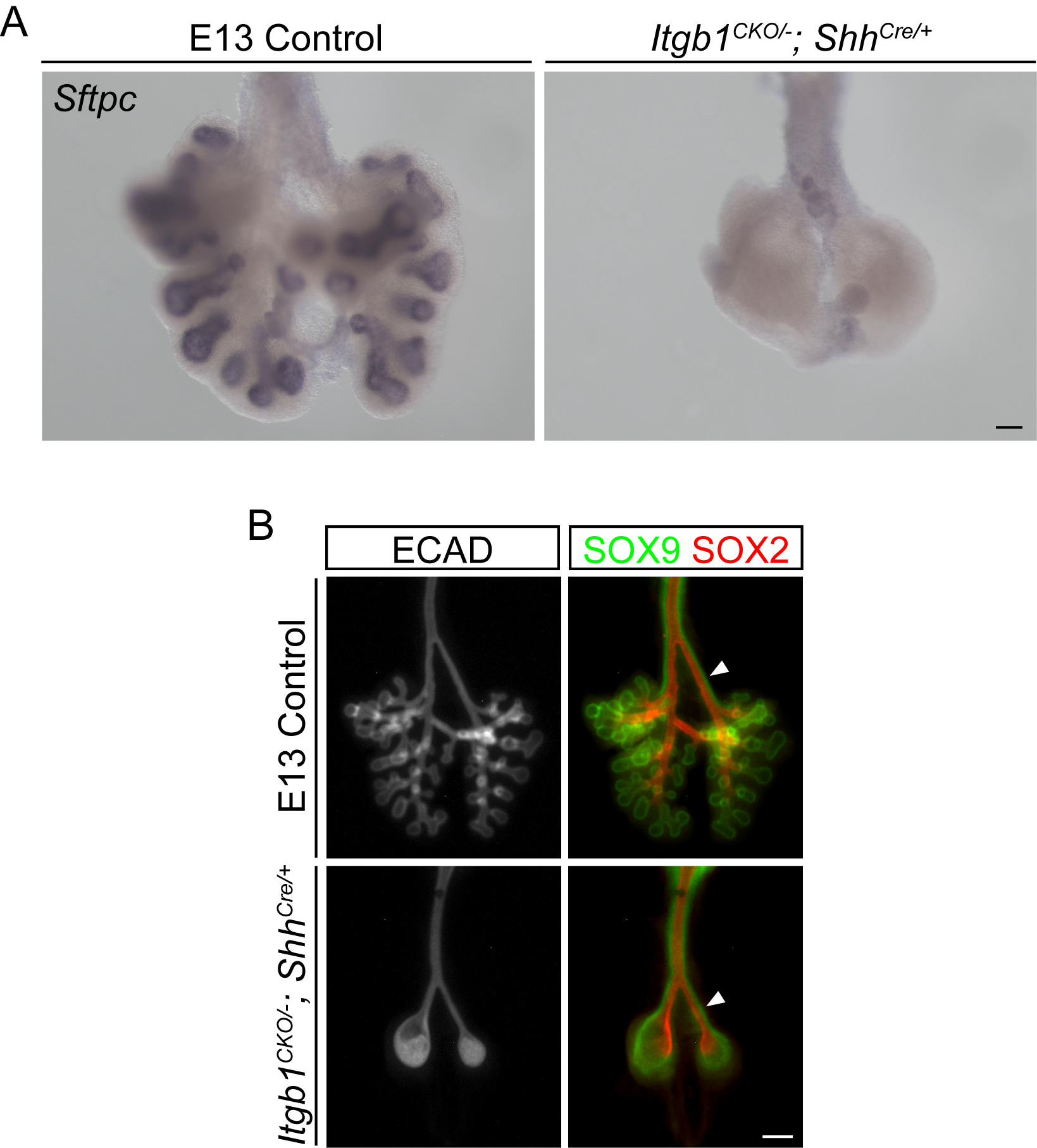

Supplement: Figure S2 — (A) Whole-mount in situ hybridization of E13 lungs shows that the Itgb1 mutant lung does not express Sftpc, a gene restricted to the branching regions of the control lung. Scale bar, 100 um. (B) Whole-mount immunostaining of E-Cadherin (ECAD), SOX9 and SOX2 of E13 lungs showing normal distribution of distal (SOX9) and proximal (SOX2) epithelial markers in the Itgb1 mutant lung. The mesenchymal SOX9 staining surrounding the extra-pulmonary airways (arrowhead) is from cartilage precursor cells. Scale bar, 200 um. (TIF) [file pone.0052886.s002.tif]

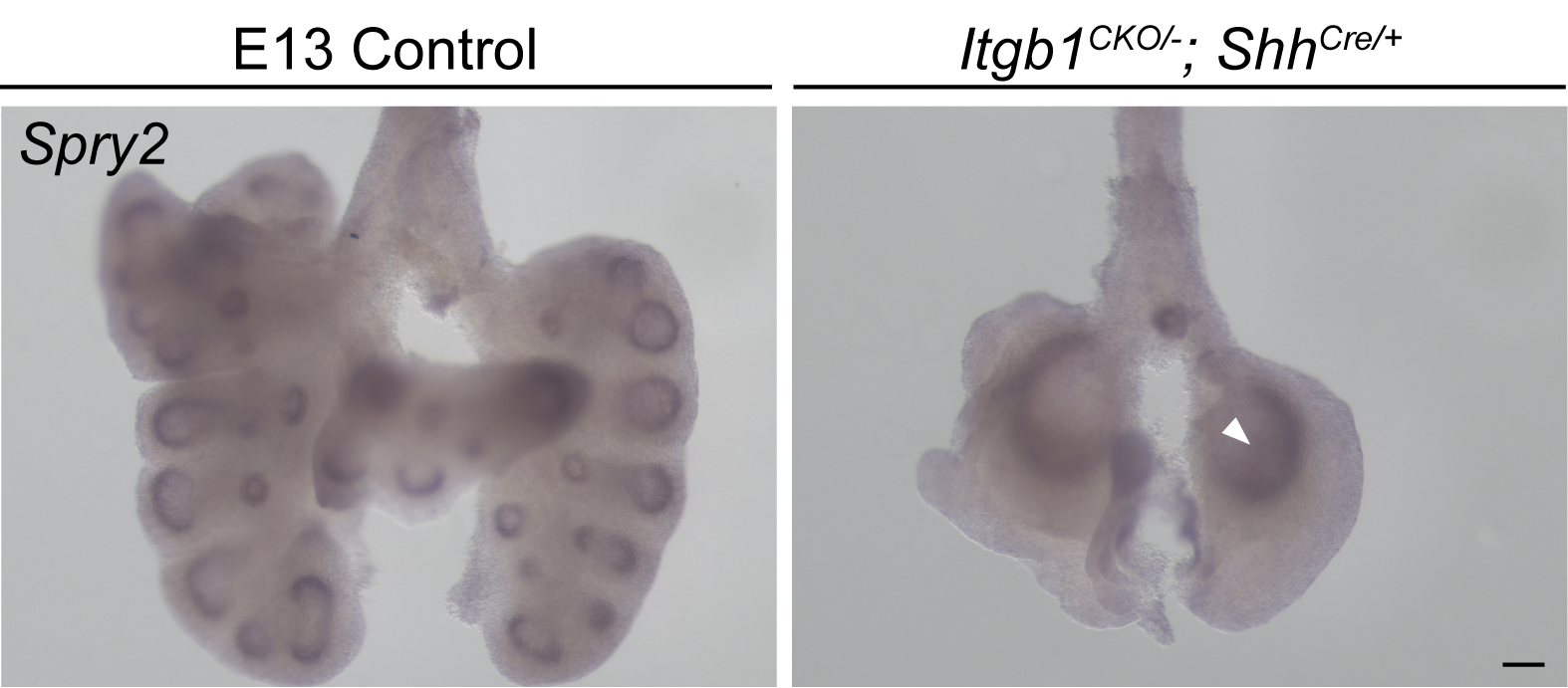

Supplement: Figure S3 — Whole-mount in situ hybridization of Spry2 in E13 lungs. Expression of Spry2 is restricted to the distal branching epithelium in the Itgb1CKO/−; ShhCre/+ mutant, like that of Bmp4 (Figure 4A), Cells on the lumenal side (arrowhead) of the multilayer mutant epithelium do not express Spry2. Scale bar, 100 um. (TIF) [file pone.0052886.s003.tif]

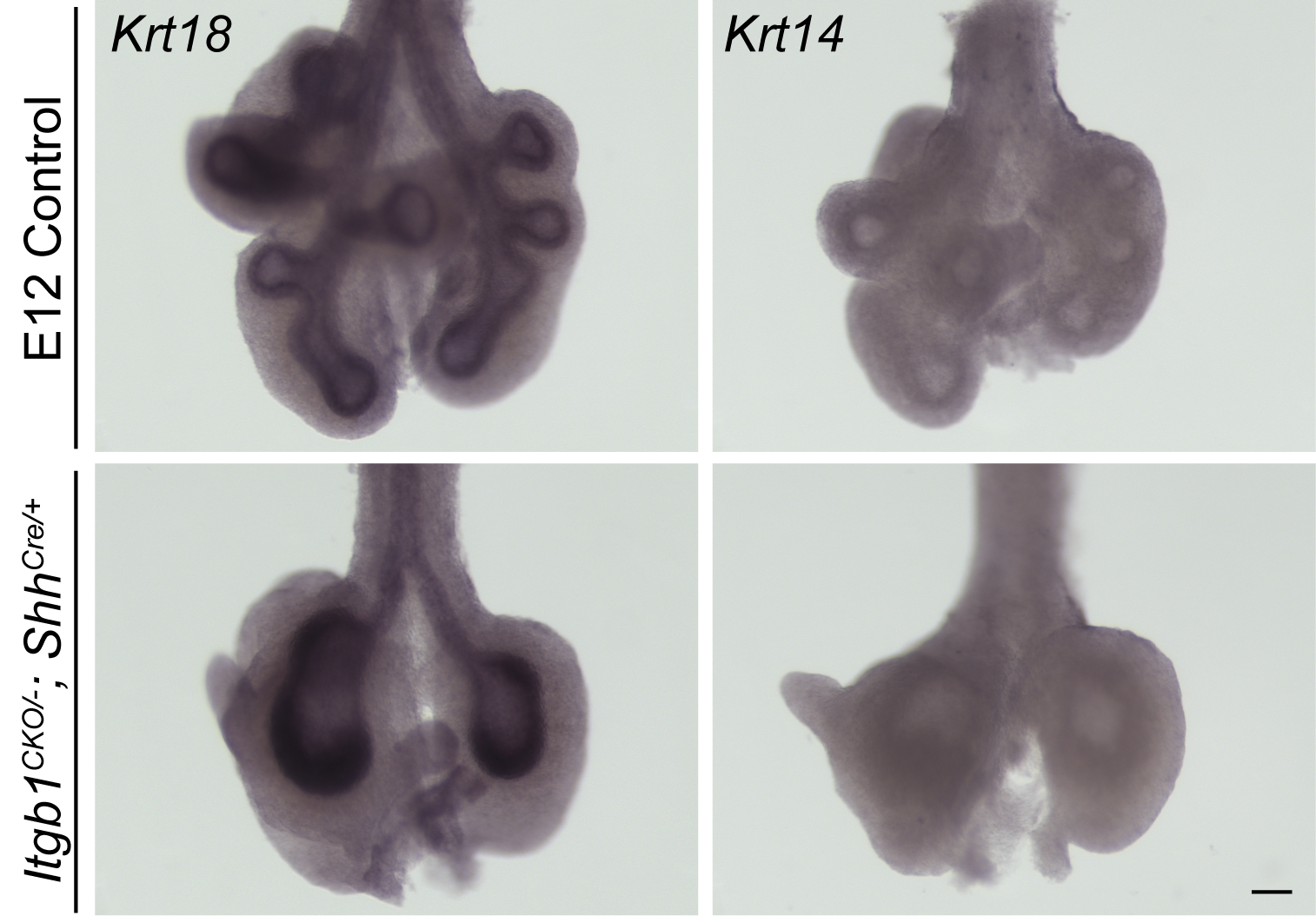

Supplement: Figure S4 — Whole-mount in situ hybridization shows that the epithelia of both control and Itgb1CKO/−; ShhCre/+ mutant lungs express a marker of simple epithelium ( Krt18 ), but not a marker of stratified epithelium ( Krt14 ) at E12. Scale bar, 100 um. (TIF) [file pone.0052886.s004.tif]
